# Supplementary material for: Genetic and clinical analyses of psychosis spectrum symptoms in a large multiethnic youth cohort reveal significant link with ADHD
Source: Transl Psychiatry. 2021 Jan 28;11:80. doi: 10.1038/s41398-021-01203-2 (PMC7844241; doi:10.1038/s41398-021-01203-2)
Supplement: Supplementary file 1 — Supplementary Figure and Table legends [file 41398_2021_1203_MOESM1_ESM.docx]

**Supplementary Figure legends**

**Figure 1.** A) Relative admixture ancestry components (based on K=3) for the EA and AA subjects in the PNC B) Cross-validation error for different ancestry components K.

**Figure 2.** MDS components based on a set of high quality independent SNPs. Orange indicates individuals assigned AA ancestry, light blue EA and dark blue are subjects with >95% EA, used for heritability analyses.

**Figure 3.** Identity by state estimates >0.05 between EA and AA pairs estimated from analysis including the whole cohort that were not identified in within-ancestry analyses. Nearly all pairs (~98%) are pairs of AA subjects.

**Figure 4.** Mean regression coefficients for the multivariate PGS based on different GWAS in All, EA and AA subjects. Standard errors indicate standard errors of the mean. Actual data is displayed on the left, models based on case-control permutations on the right.

**Figure 5.** Standardized ADHD PGS in youth with PS versus non-PS youth for All, EA, and AA.

**Figure 6.** AUCROC for each elastic net model trained on 70% and tested on the remaining 30% of data in All (pink), EA (blue) and AA (orange). Box plots indicate the median and the lower and upper hinges correspond to the first and third quartiles. The grey dots and boxplots here refer to a model including only the ADHD PGS within each ancestry group.

**Figure 7.** Association of ADHD PGS with PS in EA varying the P-value threshold.

**Figure 8.** Correlation between ADHD PGS based on the entire sample and the European-only sample.

**Figure 9.** Spearman rank correlations between PRIME and SOPS psychosis scores and Inattention and hyperactivity ADHD scores in EA, AA, and All samples.

**Figure 10.** Relation between Inattention and Hyperactivity scores and proportion of PS cases in EA, AA, and All samples.

**Figure 11.** Relation between PRIME and SOPS scores and proportion of ADHD cases in EA, AA, and All samples.

**Figure 12.** Inattention (a), Hyperactivity (b), PRIME (c) and SOPS (d) scores by case category: neither (subjects that are not ADHD or PS cases), ADD_only (ADHD cases that are not PS cases), PS_only (PS cases that are not ADHD cases), both (subjects that are both PS and ADHD cases), in EA, AA, and All samples.

**Supplementary Table legends**

**Table 1. Summary of GWAS used for analyses**

For summary statistics not available through LD-hub, we estimated the heritability on the liability scale and other GWAS parameters by running LD score regression(17), using the population prevalence as reported in the GWAS.

**Table 2.** **Univariate association of ADHD PGS with PS across ancestry groups**, correcting for phenotypic overlap and substance use, where: 95 subjects with >95% EA; noADHD removing ADHD subjects; noADD011 removing subjects that answer “yes” to the question ADD011 “Did you often have trouble paying attention or keeping your mind on your school, work, chores, or other activities that you were doing?”; Hyperactivityscore is the Hyperactivity score, InattentionScore is the Inattention score; noSymptoms removing subjects that endorse any of the symptoms in the ADHD screener; Substances: OTC over the counter, ALC alcohol, TOB tobacco, MAR marihuana, COC cocaine. Other phenotypes include Depression, Mania, Anxiety and Trauma (defined in supplemental note)

**Table 3.** **Clinical overlap between PS and other phenotypes** across ancestry groups. Confusion matrices of overlap between PS and phenotypes related to Depression, Mania, Anxiety and Trauma (as defined in supplemental note). Colors indicate different ancestry groups All pink, EA blue, AA Orange.

|  | |  | PS_ALL |  | PS_EA |  | PS_AA |  |
| --- | --- | --- | --- | --- | --- | --- | --- | --- |
|  | |  | 1 | 0 | 1 | 0 | 1 | 0 |
| Depression | | 1 | 596 | 935 | 314 | 681 | 224 | 184 |
|  | | 0 | 767 | 4761 | 389 | 3374 | 301 | 1034 |
|  | | Overlap Statistic | OR 4.0 (3.5-4.5)  P<2.2*10^-16^ | | OR 4.0 (3.4-4.8) P<2.2*10^-16^ | | OR 4.2 (3.3-5.3) P<2.2*10^-16^ | |
|  | |  |  |  |  |  |  |  |
| Mania | | 1 | 580 | 799 | 303 | 540 | 228 | 204 |
|  | | 0 | 784 | 4894 | 400 | 3511 | 299 | 1015 |
|  | | Overlap Statistic | OR 4.5 ( 4.0-5.2), P<2.2*10^-16^ | | OR 4.9 (4.1-5.9), P<2.2*10^-16^ | | OR 3.8 (3.0-4.8), P<2.2*10^-16^ | |
|  | |  |  |  |  |  |  |  |
| Anxiety | | 1 | 591 | 1486 | 333 | 1159 | 198 | 220 |
|  | | 0 | 776 | 4215 | 373 | 2901 | 328 | 998 |
|  | | Overlap Statistic | OR 2.2 (1.9-2.4), P<2.2*10^-16^ | | OR 2.2 (1.9-2.6) P<2.2*10^-16^ | | OR 2.7 (2.2-3.5) P<2.2*10^-16^ | |
|  |  | |  |  |  |  |  |  |
| Trauma | 1 | | 577 | 1219 | 276 | 753 | 244 | 359 |
|  | 0 | | 787 | 4464 | 427 | 3297 | 282 | 853 |
|  | Overlap Statistic | | OR 2.7 (2.4-3.0), P<2.2*10^-16^ | | OR 2.8 (2.4-3.4) P<2.2*10^-16^ | | OR 2.0 (1.7-2.6) 2.6 *10^-11^ | |
